# Growth Differentiation Factor 15 as a Biomarker of Cardiovascular Burden and Mortality in a Population-Based Cohort

**DOI:** 10.3390/ijms27073078

**Published:** 2026-03-27

**Authors:** Beatriz Martín-Carro, Leticia Nieto-García, Clara Sánchez-Pablo, Alfonso Romero, Candelas Pérez del Villar, José Carlos Moyano-Maza, José María de Dios, David Cembrero-Fuciños, Estefanía Iglesias-Colino, Paz Muriel, Sara Cascón, Amalia Martín-Gallego, Baltasara Blázquez, Inmaculada Santolino, Lydia González-González, María Concepción Ledesma, Javier Maillo-Seco, Jesús Rodríguez-Nieto, Luis M. Rincón, María Isidoro-García, Pedro L. Sánchez

**Affiliations:** 1 Department of Cardiology, University Hospital of Salamanca, 37007 Salamanca, Spain; 2 Instituto de Investigación Biomédica de Salamanca (IBSAL), 37007 Salamanca, Spain; 3 Centro de Investigación Biomédica en Red de Enfermedades Cardiovasculares (CIBER-CV), Instituto de Salud Carlos III, 28029 Madrid, Spain; 4 Miguel Armijo Primary Care Centre, 37007 Salamanca, Spain; 5 Faculty of Medicine, University of Salamanca, 37007 Salamanca, Spain; 6 Clinical Biochemistry Department, University Hospital of Salamanca, 37007 Salamanca, Spain; 7 Periurbana Norte Primary Care Center, 37184 Salamanca, Spain; 8 La Alamedilla Primary Care Centre, 37003 Salamanca, Spain; 9 Robleda Primary Care Center, 37521 Salamanca, Spain; 10 Miranda del Castañar Primary Care Centre, 37660 Salamanca, Spain; 11 Santa Marta Primary Care Centre, 37900 Salamanca, Spain; 12 Peñaranda de Bracamonte Primary Care Centre, 37300 Salamanca, Spain

**Keywords:** GDF15, cardiovascular disease, multimorbidity, mortality, population-based cohort

## Abstract

Growth differentiation factor 15 (GDF15) is a stress-responsive cytokine strongly associated with aging, multimorbidity, and cardiovascular disease. Although prior studies have established its prognostic value in high-risk populations, its role in the general population remains less defined. The aim of this study was to determine if there is an association between plasma GDF15 levels, heart disease and mortality in a representative population-based cohort. We analyzed 1532 participants (mean age 55 years; 54.6% women) with available baseline plasma GDF15 concentrations. Participants were stratified according to an optimal cutoff of 1081 pg/mL, derived from ROC curve analysis for mortality. Associations with prevalent heart disease were assessed using multivariable logistic regression models adjusted for cardiovascular risk factors and NT-proBNP. Mortality was analyzed using Cox proportional hazards models, with model performance evaluated by C-index and time-dependent ROC curves. Individuals with GDF15 > 1081 pg/mL were older and exhibited a more adverse cardiometabolic profile with higher prevalence of comorbidities. Elevated GDF15 was independently associated with ischemic cardiomyopathy (OR 3.34, 95% CI: 1.38–8.11), particularly in men (OR 4.26, 95% CI: 1.40–12.96), but not in women. No independent associations were observed with arrhythmias, valvulopathy, or heart failure after adjustment for NT-proBNP. During a median follow-up of 6.2 years, 51 deaths occurred. Elevated GDF15 independently predicted all-cause mortality (HR 2.47, 95% CI: 1.19–5.13), though the effect was attenuated after adjustment for NT-proBNP. GDF15 improved model discrimination (ΔC-index = +0.01; LRT *p* = 0.011) and showed robust time-dependent predictive ability, with AUCs of 0.76, 0.82, and 0.85 at 2, 4, and 6 years, respectively. In this population-based cohort, elevated GDF15 identified individuals with an adverse health profile, was independently associated with ischemic cardiomyopathy in men, and predicted mortality. Although its incremental predictive value over NT-proBNP was modest, GDF15 could provide complementary biological information and may enhance multimarker strategies for cardiovascular risk stratification in the general population.

## 1. Introduction

Cardiovascular diseases remain the leading cause of death worldwide, representing a major global health challenge [1,2]. Despite significant advances in diagnosis and treatment, the early identification of individuals at high risk for adverse cardiovascular events remains an unmet need. In this context, circulating biomarkers have attracted increasing attention as tools to refine risk prediction and support personalized prevention strategies.

Growth differentiation factor 15 (GDF15), initially known as macrophage inhibitory cytokine-1 (MIC-1), belongs to the transforming growth factor-β (TGF-β) superfamily, although it does not signal through classical TGF-β receptors [3]. Under physiological conditions, its expression is low in most tissues except the placenta, where levels are higher due to its role in supporting fetal growth and modulating immune tolerance during pregnancy. Circulating levels usually range between 0.15 and 1.15 ng/mL [4,5]. However, GDF15 concentrations rise sharply in response to cellular stressors such as mitochondrial dysfunction, inflammation, ischemia, and tissue injury [6]. It can be secreted by macrophages, vascular smooth muscle cells, endothelial cells, and adipocytes [7,8], with major circulating sources including the liver, lungs, colon, heart, and kidneys [9]. Beyond its role as a stress-response cytokine, GDF15 is strongly upregulated with aging and has been associated with multimorbidity and age-related disorders [10].

Circulating GDF15 levels are modulated by multiple clinical and lifestyle factors, increasing progressively with age and in individuals with chronic kidney disease [11], metabolic disorders such as type 2 diabetes and obesity [4,12], chronic infections [13], cancer [12,14], and in smokers [15]. This broad range of determinants highlights the need for careful interpretation of GDF15 values in clinical and epidemiological settings.

The biological effects of GDF15 are mainly mediated through the glial-derived neurotrophic factor (GDNF) receptor alpha-like (GFRAL), primarily expressed in the brainstem [16]. Upon binding to the receptor, the GDF15-GFRAL complex binds and phosphorylates the co-receptor tyrosine kinase RET, a tyrosine kinase that activates downstream signalling cascades including the AKT, ERK1/2, and PKC pathways [17]. Activation of the GDF15-GFRAL pathway has been linked to appetite regulation, energy homeostasis, and systemic stress responses, further extending the potential clinical relevance of this cytokine [18]. However, the precise stimuli that increase GDF15 and the mechanisms underlying its diverse effects remain poorly understood.

GDF15 exhibits favorable preanalytical characteristics, allowing reliable quantification in serum and plasma by immunoassay [19]. Numerous studies have associated elevated GDF15 concentrations with a wide spectrum of cardiac disorders [20], including myocardial hypertrophy [21], coronary artery disease [22], atrial fibrillation [23] and heart failure [24]. Notably, GDF15 independently predicts adverse cardiovascular events and mortality in both chronic and acute settings [25]. In heart failure, regardless of ejection fraction, higher GDF15 concentrations correlate with worse prognosis and increased mortality [26]. Similarly, in coronary artery disease, GDF15 predicts ischemic events and long-term mortality beyond traditional risk factors [27]. Evidence also supports its utility in risk prediction for valvular heart disease [28] and peripheral artery disease [29].

However, most previous studies have been conducted in high-risk or hospital-based populations, limiting the generalizability of findings to the general population. The SALMANTICOR cohort [30], a representative population-based study from Salamanca, Spain, provides a valuable opportunity to address this gap. With detailed clinical, biochemical, lifestyle, socioeconomic and cardiac imaging data, it enables comprehensive cardiovascular risk assessment in the general population.

The present study aimed to evaluate the association between plasma GDF15 concentrations, heart disease, and mortality in this representative population-based cohort. The central hypothesis was that higher GDF15 levels independently predict adverse outcomes and add incremental prognostic information beyond established cardiovascular risk factors. Findings from this analysis could support the integration of GDF15 into cardiovascular risk assessment and help inform more personalized prevention strategies at the population level.

## 2. Results

### 2.1. Baseline Characteristics

A total of 1532 participants (837 women and 695 men) with available baseline growth differentiation factor 15 (GDF15) measurements were included in the analysis. The mean follow-up was 6.1 years (median: 6.2 years).

Baseline characteristics stratified by GDF15 levels (≤1081 pg/mL vs. >1081 pg/mL) are summarized in Table 1. The overall median age was 55 years [interquartile range (IQR): 43–67], and 54.6% were women. Individuals with GDF15 > 1081 pg/mL were significantly older (median 72 (62–79) vs. 51 (40–61) years, *p* < 0.001).

The proportion of men was significantly higher in the >1081 pg/mL group compared with the ≤1081 pg/mL group (52.2% vs. 43.4%), whereas women were less frequently represented in the high-GDF15 category (47.8% vs. 56.6%; *p* < 0.01). When age distribution was analyzed separately by sex, participants with GDF15 > 1081 pg/mL showed a clear shift toward the highest age category in both women and men.

#### Adverse Profile and Comorbidities Associated with Elevated GDF15

The high GDF15 group exhibited a more adverse anthropometric and hemodynamic profile, with greater body weight, waist circumference, and body mass index (BMI), higher systolic and diastolic blood pressure and heart rate, and lower height and oxygen saturation (Table 1).

The high GDF15 group had a higher prevalence of hypertension (64.3%), diabetes (31.1%), and dyslipidemia (69.7%), accompanied by increased fasting glucose, HbA1c, and triglycerides, and lower HDL, LDL, and total cholesterol (all *p* < 0.001). These findings were consistent with the greater use of glucose-lowering (17.6%) and lipid-lowering therapies (40.3%).

Comorbidities were more frequent among individuals with GDF15 > 1081 pg/mL, including kidney failure, cerebrovascular disease, peripheral/aortic/carotid vascular disease, chronic obstructive pulmonary disease (COPD), and cancer (all *p* < 0.05). Neuropsychiatric conditions (dementia, depression) were also more common, while anemia and asthma were more prevalent in the low GDF15 group. Lifestyle differences were also observed: daily alcohol consumption was more frequent in the high GDF15 group (31.4% vs. 24.0%, *p* = 0.006), and physical inactivity was more common (15.3% vs. 11.2%). Adherence to the Mediterranean diet showed only minor, non-clinically relevant differences.

Biochemical analyses showed significantly higher levels of N-terminal pro-brain natriuretic peptide (NT-proBNP), troponin I, creatinine, aspartate transaminase (AST), glycated hemoglobin, and high-sensitivity C-reactive protein (hs-CRP) among individuals with elevated GDF15.

### 2.2. Association of GDF15 with Heart Diseases

**Figure 1:**
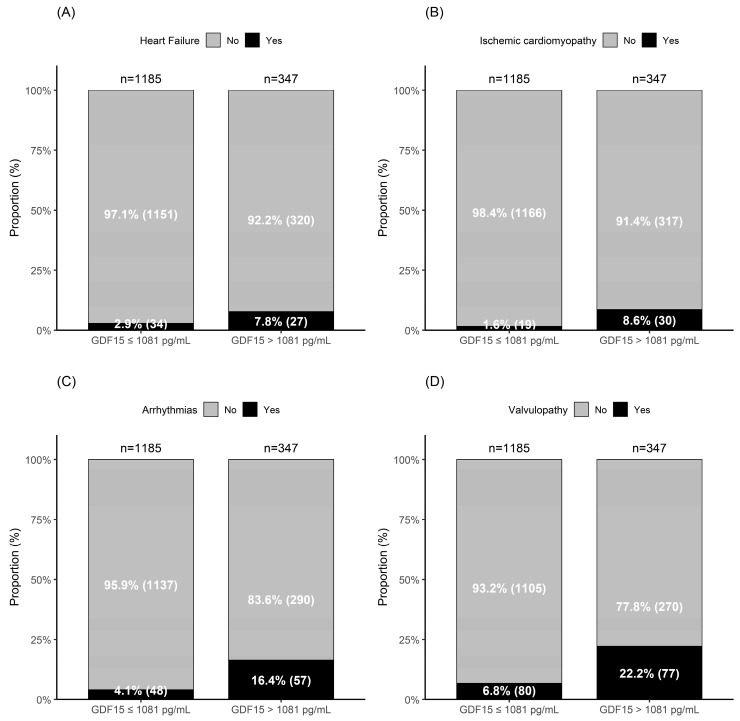
Prevalence of baseline heart disease according to GDF15 group (≤1081 pg/mL vs. >1081 pg/mL): (**A**) Heart failure, (**B**) Ischemic cardiomyopathy, (**C**) Arrhythmias, and (**D**) Valvulopathy. All *p* < 0.001.

At baseline, 275 participants (18%) had prevalent heart disease, defined as heart failure (n = 61, 4.0%), ischemic cardiomyopathy (n = 49, 3.2%), arrhythmias (n = 105, 6.9%), and/or valvulopathy (n = 157, 10.3%). These categories were not mutually exclusive, and some participants presented more than one heart disease (Figure 1).

The prevalence of heart disease was significantly higher in the high GDF15 group compared with the low GDF15 group (34.6% [n = 120] vs. 13.1% [n = 155], *p* < 0.001). Each individual condition, heart failure (Figure 1A), ischemic cardiomyopathy (Figure 1B), arrhythmia (Figure 1C), and valvulopathy (Figure 1D) was more prevalent in the high GDF15 group (all *p* < 0.001).

#### Logistic Regression Models for Baseline Heart Disease

Logistic regression models were performed to evaluate the association of GDF15 with specific baseline heart disease (Model 1: adjusted for age, sex, current smoking, daily alcohol consumption, BMI; Model 2: fully adjusted: model 1 plus NT-proBNP). Results are summarized in Table 2 and Table S1.

In unadjusted analyses, GDF15 > 1081 pg/mL was strongly associated with heart failure (OR 2.86; 95% CI: 1.70–4.82), ischemic cardiomyopathy (OR 5.85; 95% CI: 3.25–10.53), arrhythmias (OR 4.66; 95% CI: 3.11–6.98), and valvulopathy (OR 2.25; 95% CI: 1.40–3.61) (all *p* < 0.001).

After adjustment for age, sex, smoking status, daily alcohol consumption, and BMI (Model 1), GDF15 remained significantly associated with heart failure (OR 1.97; 95% CI: 1.03–3.78; *p* = 0.040) and ischemic cardiomyopathy (OR 2.43; 95% CI: 1.20–4.91; *p* = 0.014), but not with arrhythmias or valvulopathy.

In all participants, in the fully adjusted model (Model 2), which included NT-proBNP, high GDF15 levels (>1081 pg/mL) were independently associated with ischemic cardiomyopathy (OR 3.34; 95% CI: 1.38–8.11; *p* = 0.008), compared with plasma GDF15 levels of ≤1081 pg/mL. High plasma GDF15 (>1081 pg/mL) remained significantly associated with ischemic cardiomyopathy in men (OR 4.26; 95% CI: 1.40–12.96; *p* = 0.011), but this was not the case for women. For heart failure, arrhythmias, and valvulopathy, NT-proBNP exhibited stronger associations, while GDF15 lost statistical significance (Table S1).

### 2.3. Mortality Outcomes

During follow-up, 51 deaths were recorded, corresponding to an overall all-cause mortality rate of 5.43 per 1000 person-years. Mortality was higher in men (8.04 per 1000 person-years) compared with women (3.29 per 1000 person-years). Cardiovascular mortality showed a similar pattern (overall: 1.38 per 1000 person-years; men: 1.89; women: 0.96).

The distribution of causes of death according to GDF15 group and sex is shown in Table 3. Overall, the most frequent causes of death were neoplasms (33.3%) and cardiovascular diseases (25.5%).

Among deaths in women (*n* = 17), the most frequent cause in those with GDF15 > 1081 pg/mL was cardiovascular disease (5 deaths, 29.4%), followed by neoplasms (2 deaths, 11.7%). No cardiovascular deaths were observed in women with GDF15 ≤ 1081 pg/mL.

Among deaths in men (*n* = 34), the most frequent cause in those with GDF15 > 1081 pg/mL was neoplasms (8 deaths, 23.5%), followed by cardiovascular disease (5 deaths, 14.7%). In men with GDF15 ≤ 1081 pg/mL, deaths due to neoplasms and cardiovascular disease occurred with similar frequency (3 deaths each, 8.8%).

In multivariate model adjusted for classical risk factors (Model A: age, sex, current smoking, daily alcohol consumption, BMI, and prevalent heart disease), elevated GDF15 remained independently associated with all-cause mortality (HR 2.47; 95% CI: (1.19–5.13), *p* = 0.015). However, after the inclusion of NT-proBNP (Model B), the association of GDF15 with all-cause mortality was attenuated, reaching borderline significance (HR 2.61; 95% CI: (0.99–6.87), *p* = 0.051), whereas NT-proBNP retained significance (HR 1.11; 95% CI: (1–1.22), *p* = 0.044) (Table S2).

### 2.4. Discriminative Performance of GDF15 and NT-proBNP

**Figure 2:**
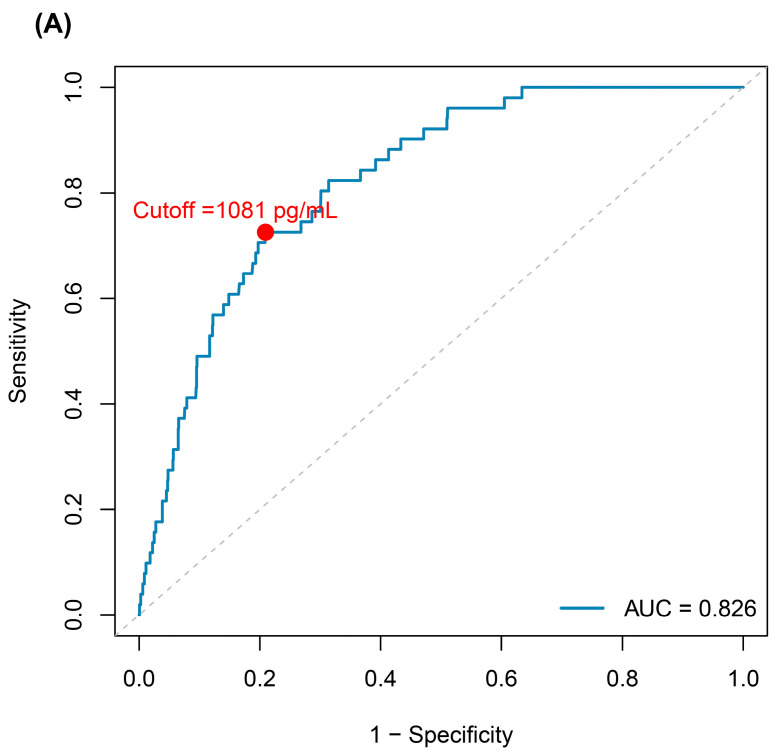

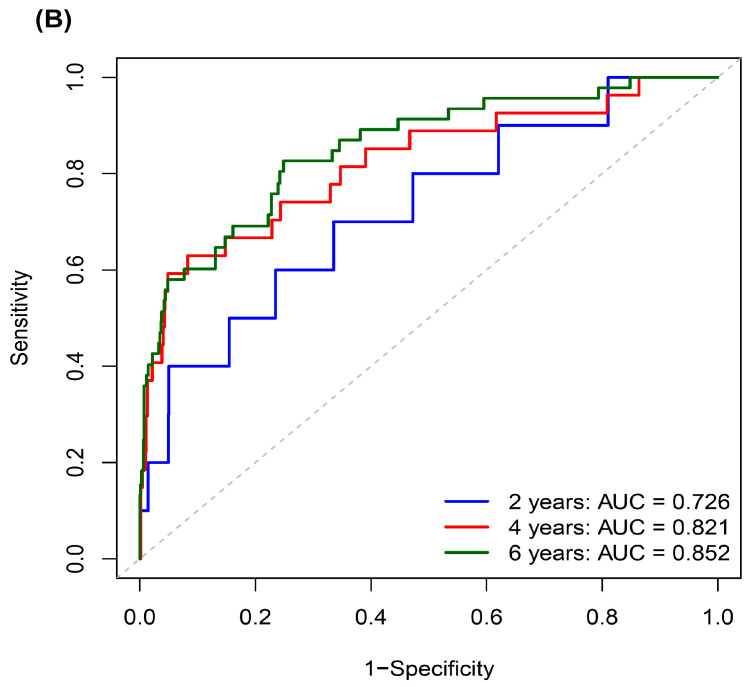
Discriminative performance of GDF15 for all-cause mortality. (**A**) Receiver operating characteristic (ROC) curve for GDF15 with AUC and optimal cutoff (1081 pg/mL) by Youden index. (**B**) Time-dependent ROC curves from Cox regression models adjusted for age, sex, smoking, alcohol, BMI, and heart disease, with AUCs at 2, 4, and 6 years. Grey dashed line indicates no-discrimination reference (AUC = 0.5).

ROC curve analysis showed good discriminative ability of GDF15 for all-cause mortality (AUC 0.826; 95% CI: 0.77–0.87). The Youden index identified 1081 pg/mL as the optimal cutoff (sensitivity 0.72; specificity 0.79) (Figure 2A).

Cox proportional hazards models further demonstrated the incremental value of GDF15 (Table 4).

The base Cox regression model including age, sex, current smoking, daily alcohol consumption, BMI, and heart disease showed a C-index of 0.839. After the addition of GDF15 (Model A), the C-index increased to 0.849 (ΔC-index = +0.01). The likelihood ratio test indicated an improvement in model fit (χ^2^ = 6.38, *p* = 0.011). In contrast, when NT-proBNP was added to the base model (Model C), the C-index increased only marginally from 0.879 to 0.880 (ΔC-index = +0.001), and the improvement in model fit did not reach statistical significance (χ^2^ = 3.76, *p* = 0.052).

Time-dependent ROC analyses confirmed robust discriminative performance of GDF15 over time, with AUCs of 0.756, 0.818, and 0.848 at 2, 4, and 6 years, respectively (Figure 2B). Pairwise comparisons of AUCs showed no significant differences across time points (all *p* > 0.05).

## 3. Discussion

In this population-based cohort, elevated plasma growth differentiation factor 15 (GDF15) levels (>1081 pg/mL) were associated with an adverse cardiometabolic and comorbidity profile, ischemic cardiomyopathy, and increased risk of all-cause mortality. These associations remained significant after adjustment for traditional risk factors, and the prognostic performance of GDF15 was comparable to that of N-terminal pro-brain natriuretic peptide (NT-proBNP). These results extend prior evidence, largely derived from hospital-based or high-risk cohorts, by showing the prognostic value of GDF15 in the general population.

GDF15 is not a disease-specific marker; instead, it reflects a broad burden of comorbidities and pathophysiological stressors, including inflammation, mitochondrial dysfunction, infection, and cancer [31]. This pleiotropic expression likely explains its strong association in our cohort with chronic kidney disease [17], diabetes [32,33], pulmonary disease [34], and vascular disorders [29,35], in addition to cardiovascular disease [17]. Prior studies have proposed GDF15 as an integrative marker of biological aging and frailty [36,37], reflecting systemic stress and cumulative multimorbidity.

The broad responsiveness of GDF15 may therefore allow it to capture biological processes contributing to cardiovascular risk that are not fully reflected by traditional cardiovascular biomarkers. This property may represent both a strength and a limitation: while it may reflect underlying systemic processes associated with cardiovascular risk, its lack of disease specificity may limit its direct clinical interpretability. Clinical studies have shown that GDF15 predicts adverse outcomes across diverse cardiovascular conditions. For example, in the PROSe-ICD study, elevated GDF15 levels predicted hospitalization and mortality in patients with heart failure but were not related to ventricular arrhythmias, suggesting that GDF15 may primarily reflect systemic biological stress rather than arrhythmic propensity [38].

Consistent with this concept, in our study elevated GDF15 levels were descriptively associated with several comorbid conditions. However, after multivariable adjustment including NT-proBNP, the independent association persisted for ischemic cardiomyopathy and was observed in men but not in women. This finding suggests potential sex-related differences in the clinical significance of GDF15 and indicates that, beyond reflecting overall health burden, GDF15 may also capture pathophysiological processes related to ischemic cardiac injury in certain subgroups.

Experimental studies support a mechanistic role for GDF15 in cardiac remodeling and injury. Cardiomyocytes, endothelial cells, vascular smooth muscle cells, and macrophages secrete GDF15 in response to oxidative stress, ischemia, mechanical stretch, angiotensin II, or proinflammatory cytokines [31]. Upregulation of GDF15 has been linked to hypoxia-induced apoptosis, fibrosis, ischemia, and cachexia [12,39,40]. Clinical studies in heart failure have consistently reported associations between GDF15 concentrations, symptom severity, left ventricular remodeling, and prognosis [40,41]. Likewise, elevated GDF15 levels have been described in ischemic cardiomyopathy [42], supporting a potential role of this biomarker in pathways related to myocardial injury and stress.

GDF15 has also been described as a Domain II adipokine/cytokine, a group of cardioprotective molecules that are upregulated in response to adiposity and metabolic stress. These molecules may exert protective effects by attenuating myocardial apoptosis, inflammation, hypertrophy, and fibrosis. However, obesity and visceral adiposity may induce resistance to the biological actions of GDF15, potentially through receptor downregulation or adipocyte mitochondrial dysfunction. In this context, elevated circulating GDF15 levels may initially reflect a compensatory response to cardiometabolic stress, whereas persistent increased levels could indicate failure of this counter-regulatory pathway and the presence of more advanced tissue injury. Consistent with this hypothesis, increased GDF15 concentrations have been detected years before the onset of overt heart failure and have been associated with subclinical cardiac fibrosis and abnormalities in diastolic filling. [8].

A novel finding of our study was the sex-specific association between elevated GDF15 levels and ischemic cardiomyopathy, which was observed only in men. Previous studies have rarely explored sex-stratified associations, and most analyses have been performed in mixed populations. Several explanations may contribute to this difference. First, men in our cohort exhibited a higher prevalence of traditional atherosclerotic risk factors (high blood pressure, high cholesterol, smoking, diabetes, hypertension, dyslipidemia, a sedentary lifestyle, and an unhealthy diet), which may interact with GDF15-driven pathways of vascular injury and ischemia. Second, hormonal and metabolic differences could modulate the expression or downstream signaling of GDF15, as sex hormones are known to influence inflammatory and mitochondrial stress responses [43]. Third, women may develop ischemic heart disease through distinct mechanisms, such as microvascular dysfunction, which may not be fully captured by circulating GDF15 levels. These findings highlight the importance of considering sex as a biological variable in biomarker research.

The present findings also reinforce the prognostic significance of GDF15 for mortality in the general population. Elevated GDF15 levels were associated with more than a two-fold increase in all-cause mortality, even after adjusting for age, sex, lifestyle, and prevalent heart disease. While this association was attenuated after accounting for NT-proBNP, suggesting that the prognostic information conveyed by these biomarkers may partly overlap, GDF15 provided incremental discrimination in risk models. However, the increase in the C-index was small, suggesting that the additional discriminative value of GDF15 beyond established predictors such as NT-proBNP may be limited.

The clinical implications of these findings should therefore be interpreted cautiously. Although GDF15 may provide complementary information reflecting systemic stress and multimorbidity, its incremental predictive value beyond established cardiac biomarkers such as NT-proBNP was modest in our study. Before its incorporation into clinical practice can be considered, further research is needed to confirm its additional prognostic value through external validation in independent cohorts and to demonstrate that its measurement results in clinically meaningful improvements in patient management and outcomes. If these conditions are met, future studies should also address the cost-effectiveness of incorporating GDF15 into routine cardiovascular risk assessment.

Several limitations should be acknowledged. First, the relatively small number of mortality events may have limited the statistical power of multivariable analyses and contributed to the wide confidence intervals observed in some models. Second, the GDF15 cutoff value was derived and internally validated within this cohort; its applicability to other populations remains uncertain, and overfitting cannot be excluded. Additionally, GDF15 was measured at a single time point, limiting assessment of longitudinal changes and dynamic risk prediction. Repeated measurements in future studies may improve the robustness of prognostic evaluation.

Another limitation relates to the availability of biological samples. Approximately 26% of participants in the original SALMANTICOR cohort lacked GDF15 measurements and were therefore excluded from the present analyses. Blood sampling in this study was linked to a genetic research component, and some participants declined this procedure despite consenting to the clinical assessment. Participation in biological sampling is known to vary across populations in epidemiological studies and may be influenced by several factors, including age, sociocultural factors, or concerns related to genetic analyses. In our cohort, individuals without available GDF15 measurements tended to be older (median age: 61 (47–74) years vs. 55 (43–67) years) and were more frequently from rural areas (191 (32.2%) vs. 340 (23.1%)), which could reflect differences in willingness to undergo blood sampling. Although the proportion of participants with available plasma samples in our study (74%) is comparable to that reported in other based population studies [44,45], the possibility of some degree of selection bias cannot be completely excluded.

In summary, GDF15 was associated with multimorbidity, ischemic cardiomyopathy, and mortality in this population-based cohort. Although its incremental predictive value beyond NT-proBNP was modest, GDF15 may still reflect broader systemic biological stress not fully captured by cardiac-specific biomarkers. Further studies are required to clarify its potential role within multimarker approaches for cardiovascular risk stratification and to determine its clinical utility in diverse populations.

## 4. Materials and Methods

### 4.1. Study Design and Population

SALMANTICOR (ClinicalTrials.gov identifier: NCT03429452) is a population-based cohort study conducted in the province of Salamanca, Spain. Between 2015 and 2018, 2063 adults aged ≥18 years were recruited using stratified sampling by sex, age, and geographic area. The study was conducted in accordance with the Declaration of Helsinki and approved by the Clinical Research Ethics Committee of the Salamanca Health Area (Salamanca, Spain). Ethical approval was granted in an official signed and stamped letter dated 29 September 2014. All participants provided written informed consent. The detailed protocol has been described previously [30].

For the present analysis, participants from the SALMANTICOR cohort with available baseline plasma GDF15 measurements were eligible. A total of 1532 participants (74% of the original cohort) had stored plasma samples and complete data for GDF15 determination and were therefore included. Individuals without available plasma samples or with missing GDF15 measurements at baseline were excluded.

Blood sampling in the SALMANTICOR study was linked to a genetic research component, and although all participants provided written informed consent to participate in the clinical assessment, some individuals declined blood extraction. Consequently, plasma samples were not available for all participants at baseline. Participation in biological sampling, particularly when associated with genetic research, may vary across individuals and populations in epidemiological studies [44,45]. Therefore, the present analysis was restricted to participants with available plasma samples for GDF15 determination.

**Figure 3:**
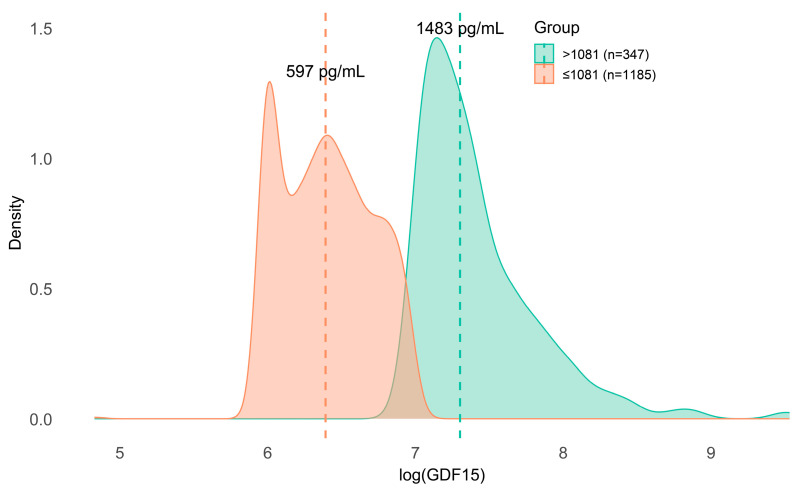
Density distribution of log-transformed plasma GDF15 according to the predefined cutoff (≤1081 pg/mL vs. >1081 pg/mL). Dashed vertical lines indicate group medians (≤1081 pg/mL: 597 pg/mL; >1081 pg/mL: 1483 pg/mL).

Individuals were stratified based on a plasma GDF15 concentration threshold of 1081 pg/mL, derived from Receiver Operating Characteristic (ROC) curve analysis for mortality using the Youden index [46]. At baseline, 1185 individuals (77.3%) had GDF15 ≤ 1081 pg/mL, and 347 (22.7%) had higher concentrations. The log-transformed GDF15 distribution showed clear separation between groups (median 597 vs. 1483 pg/mL) (Figure 3).

### 4.2. Clinical Assessment

All participants underwent a standardized clinical assessment including anthropometric measurements, electrocardiography, echocardiography, and blood sampling. Structured questionnaires were used to collect sociodemographic data, lifestyle habits (diet, alcohol consumption, smoking, and physical activity), medication use, and personal and family medical history.

Participants were followed up from their baseline visit until 31 January 2023, with a mean follow-up of 6.1 years (median: 6.2 years). During this period, no follow-up visits were conducted, and mortality was the sole longitudinal outcome.

### 4.3. Biomarkers and Laboratory Determinations

Fasting blood samples were collected after at least 12 h of fasting and abstinence from nicotine, alcohol, and caffeine. Serum and plasma were separated by centrifugation at 3500 rpm for 5 min and stored at –80 °C until analysis.

Plasma concentrations of GDF15, NT-proBNP, Ferritin, and high-sensitivity Troponin T (hs-TnT) were determined by electrochemiluminescence immunoassay (ECLIA), using ELECSYS^®^ reagents from Roche Diagnostics, (Mannheim, Germany) on a cobas^®^ e601 analytical module. The limits of detection for these assays are: 400 ng/mL for GDF-15, 5 pg/mL for NT-proBNP, 0.5 ng/mL for Ferritin and 1.3 ng/L for hs-TnT. Plasma concentrations of glucose, high-sensitivity C-reactive protein (hs-CRP), cholesterol, HDL-cholesterol, LDL-cholesterol, triglycerides, creatinine, AST, ALT, iron, transferrin, chloride and sodium and potassium ions were determined using photometric, immunoturbidimetric and indirect potentiometry with ion-selective electrodes (ISE) methods on cobas^®^ c501 analyzers from Roche Diagnostics. HbA1c determinations were performed using high-performance liquid chromatography (HPLC) on HA-8160 analyzers from Menarini Diagnostics (Florence, Italy).

All analytical determinations were performed at the Laboratory of Clinical Analysis and Clinical Biochemistry of the University Hospital of Salamanca. Calibrations and quality controls were performed according to the manufacturer’s instructions, and the determinations were carried out by laboratory personnel without access to patients’ clinical information.

### 4.4. Cardiovascular Imaging

All participants in the population-based SALMANTICOR cohort were invited to undergo a comprehensive cardiovascular examination at their respective primary healthcare center, regardless of known heart disease. Electrocardiography and transthoracic echocardiography were performed by trained cardiologists following standardized protocols. Structural and functional cardiac parameters, including left ventricular ejection fraction and diastolic function measures, were systematically recorded.

### 4.5. Outcomes

At inclusion, demographic and anthropometric data were recorded, along with cardiovascular risk factors (hypertension, diabetes mellitus, dyslipidaemia, smoking, and daily alcohol consumption), history of cardiovascular disease (stroke or transient ischemic attack (TIA), coronary artery disease, congenital heart disease, atrioventricular block, peripheral/aortic/carotid vasculopathy), and non-cardiovascular disease (anemia/bleeding, rheumatologic/immunologic disease, chronic kidney disease, cancer, COPD, asthma, cognitive impairment/dementia, depression, and anxiety). Lifestyle variables, including physical activity level and adherence to the Mediterranean diet, were also assessed.

Baseline laboratory parameters included fasting glucose, lipid profile, inflammatory markers (hs-CRP), and cardiac biomarkers (troponin I and NT-proBNP).

Mortality follow-up is ongoing and updated approximately every five years; for the present analysis, it covers the period from participants’ baseline visit until 31 January 2023 (mean follow-up 6.1 years, median 6.2 years). Deaths were ascertained through hospital records, national death registries, and primary care databases. Information on cause of death was obtained from the Spanish National Statistics Institute (INE), and cause-specific deaths were classified according to the 10th revision of the International Classification of Diseases (ICD-10).

The primary outcomes were cardiovascular mortality (all codes between I00 and I99), and non-cardiovascular mortality (other ICD-10 codes not classed as cardiovascular).

### 4.6. Statistical Analysis

Data were described using median [interquartile range (IQR)] for non-normally distributed variables and percentages for categorical variables. For comparisons between GDF15 ≤ 1081 pg/mL group and GDF15 > 1081 pg/mL group, the Mann–Whitney U test was applied for continuous variables, and the Chi-squared test for categorical variables.

Baseline associations between GDF15, as the dichotomic (≤1081 pg/mL, >1081 pg/mL) independent variable, and prevalent heart disease -defined as the presence of heart failure, ischemic cardiomyopathy, arrhythmias, and/or valvulopathy- were assessed with logistic regression. Different models were fitted:

- Unadjusted model: included GDF15 as the sole independent variable.
- Base model: adjusted by age, sex, current smoking, daily alcohol consumption, and body mass index (BMI), but excluded GDF15 and NT-proBNP.
- Model 1: base model plus GDF15
- Model 2: Model 1 plus NT-proBNP.

These independent variables were selected based on their known association with the outcomes of interest. Age, BMI and NT-proBNP were standardized to a mean of 0 and standard deviation (SD) of 1 before model fitting. This approach ensures that the reported odds ratios (ORs) correspond to an increase of 1 SD in each of these variables, thereby improving the interpretability of effect sizes. Interaction between GDF15 and sex was tested in all models. The calibration of logistic regression models was assessed using the Hosmer–Lemeshow goodness-of-fit test.

Cox proportional hazard models were fitted to analyse the association between the relative risk of death and baseline plasma GDF15. Four different multivariate models were used to adjust hazard ratios including the following variables:

- Base model: age, sex, current smoking, daily alcohol consumption, BMI, and prevalent heart disease.
- Model A: Base model + GDF15.
- Model B: Model A + NT-proBNP.
- Model C: Base model + NT-proBNP.

The effect modification of the association between plasma GDF15 and mortality by sex was assessed. Hazard ratios (HRs) and 95% confidence intervals (CIs) were reported.

Model performance was evaluated using the concordance index (C-index) to quantify discriminative ability, and the Likelihood Ratio Test (LRT) to compare nested models. The incremental predictive value of GDF15 and NT-proBNP was expressed as the change in C-index (ΔC-index) between base and extended models.

Time-dependent ROC curves were constructed, and the area under the curves (AUCs) were used to evaluate prediction at 2, 4, and 6 years.

A two-sided *p*-value < 0.05 was considered statistically significant. R software for statistical computing and graphics (version 4.4.3; R Foundation for Statistical Computing, Vienna, Austria) was used for all statistical analysis.

## 5. Conclusions

In this population-based cohort, elevated plasma GDF15 levels were associated with a higher burden of cardiovascular disease, particularly ischemic cardiomyopathy, and with an increased risk of all-cause mortality. These associations remained independent of traditional risk factors, lifestyle variables, and prevalent cardiovascular disease. Notably, the association with ischemic cardiomyopathy was observed only in men, suggesting potential sex-related differences in the clinical relevance of this biomarker.

Overall, these findings highlight the potential role of GDF15 as a biomarker for cardiovascular risk stratification in population-based settings. However, given its pleiotropic nature and the observational design of this study, further research is required to confirm its prognostic utility, clarify underlying mechanisms, and determine whether its incorporation into multimarker strategies could improve cardiovascular risk prediction and prevention.

## Figures and Tables

**Table 1:** Main baseline characteristics of participants included overall and by GDF15 categories (≤1081 pg/mL vs. >1081 pg/mL).

	Overall	GDF15 ≤ 1081 pg/mL	GDF15 > 1081 pg/mL	*p*-Value
N	1532	1185	347	
Demographic data				
Sex (%)				
Women	837 (54.6)	671 (56.6)	166 (47.8)	0.005
Men	695 (45.4)	514 (43.4)	181 (52.2)	0.005
Age (%)				<0.001
Women				
(18, 48)	306 (20.0)	295 (24.9)	11 (3.2)	
(49, 63)	274 (17.9)	240 (20.3)	34 (9.8)	
(64, 93)	257 (16.8)	136 (11.5)	121 (34.9)	
Men				
(18, 48)	233 (15.2)	224 (18.9)	9 (2.6)	
(49, 63)	233 (15.2)	192 (16.2)	41 (11.8)	
(64, 93)	229 (14.9)	98 (8.3)	131 (37.8)	
Physical examination				
Weight (kg) [median (IQR)]	70.00 (62.00, 81.00)	70.00 (61.00, 81.00)	74.00 (64.00, 82.50)	0.005
Height (cm) [median (IQR)]	165.00 (160.00, 171.00)	166.00 (160.00, 172.00)	164.00 (159.50, 169.00)	<0.001
Waist circumference (cm) [median (IQR)]	88.00 (81.00, 97.00)	87.00 (80.00, 95.12)	94.00 (86.00, 102.00)	<0.001
BMI (kg/m^2^) [median (IQR)]	25.64 (23.09, 28.70)	25.25 (22.72, 28.25)	27.04 (24.42, 30.15)	<0.001
Heart rate (bpm) [median (IQR)]	65.00 (61.00, 75.00)	65.00 (61.00, 74.00)	69.00 (61.00, 75.00)	0.021
SBP (mmHg) [median (IQR)]	137.00 (125.00, 149.00)	135.00 (124.00, 146.00)	145.00 (135.00, 157.00)	<0.001
DBP (mmHg) [median (IQR)]	86.00 (79.00, 93.00)	85.00 (79.00, 92.00)	88.00 (81.00, 94.00)	0.003
O_2_ saturation (%) [median (IQR)]	98.00 (97.00, 99.00)	98.00 (97.00, 99.00)	97.00 (96.00, 98.00)	<0.001
Risk factors and non-cardiovascular medical history				
Smoker (%)				0.155
Current-smoker	296 (19.3)	241 (20.3)	55 (15.9)	
Ex-smoker	442 (28.9)	334 (28.2)	108 (31.1)	
Non-smoker	794 (51.8)	610 (51.5)	184 (53.0)	
Daily alcohol consumption = Yes (%)	393 (25.7)	284 (24.0)	109 (31.4)	0.006
Hypertension = Yes (%)	629 (41.1)	406 (34.3)	223 (64.3)	<0.001
Antihypertensive drugs = Yes (%)	380 (25.6)	197 (17.2)	183 (53.4)	<0.001
Glucose (mg/dL) [median (IQR)]	89.00 (81.00, 99.00)	88.00 (80.00, 97.00)	94.50 (84.75, 110.00)	<0.001
Diabetes = Yes (%)	163 (10.6)	55 (4.6)	108 (31.1)	<0.001
Hypoglycemic drugs = Yes (%)	77 (5.0)	16 (1.4)	61 (17.6)	<0.001
Total cholesterol (mg/dL) [median (IQR)]	182.00 (160.00, 202.00)	184.00 (162.00, 204.00)	173.00 (152.75, 196.00)	<0.001
HDL cholesterol (mg/dL) [median (IQR)]	52.00 (43.90, 61.80)	52.70 (44.80, 62.20)	49.30 (42.00, 59.10)	0.001
LDL cholesterol (mg/dL) [median (IQR)]	105.18 (87.12, 122.94)	107.81 (88.92, 125.19)	96.64 (79.87, 114.81)	<0.001
Triglycerides (mg/dL) [median (IQR)]	98.10 (70.80, 137.30)	93.80 (67.50, 134.50)	111.10 (82.90, 155.15)	<0.001
Dyslipidemia	869 (56.7)	627 (52.9)	242 (69.7)	<0.001
Lipid-lowering drugs = Yes (%)	329 (21.5)	189 (15.9)	140 (40.3)	<0.001
History of anemia/bleeding = Yes (%)	312 (20.4)	255 (21.6)	57 (16.4)	0.045
Rheumatologic/immunologic disease = Yes (%)	26 (8.2)	95 (8.0)	31 (8.9)	0.669
Chronic kidney disease = Yes (%)	16 (1.0)	8 (0.7)	8 (2.3)	0.020
Cancer = Yes (%)	94 (6.1)	57 (4.8)	37 (10.7)	<0.001
COPD = Yes (%)	30 (2.0)	14 (1.2)	16 (4.6)	<0.001
Asthma = Yes (%)	104 (6.8)	89 (7.5)	15 (4.3)	0.050
Cognitive impairment/dementia = Yes (%)	8 (0.5)	2 (0.2)	6 (1.7)	0.002
Depression = Yes (%)	314 (20.5)	220 (18.6)	94 (27.1)	0.001
Anxiety = Yes (%)	614 (40.1)	484 (40.8)	130 (37.5)	0.286
Risk factors and cardiovascular medical history				
Stroke/TIA = Yes (%)	35 (2.3)	18 (1.5)	17 (4.9)	<0.001
Peripheral/aortic/carotid vasculopathy = Yes (%)	78 (5.1)	48 (4.1)	30 (8.7)	0.001
Coronary revascularization = Yes (%)	31 (2.0)	10 (0.8)	21 (6.1)	<0.001
Cardiomyopathy/congenital heart disease = Yes (%)	13 (0.9)	8 (0.7)	5 (1.5)	0.279
Atrioventricular block (first or second degree) = Yes (%)	73 (4.8)	41 (3.5)	32 (9.4)	<0.001
Level of physical activity				0.008
High	121 (7.9)	105 (8.9)	16 (4.6)	
Low or inactive	186 (12.1)	133 (11.2)	53 (15.3)	
Moderate	1224 (79.9)	946 (79.9)	278 (80.1)	
Mediterranean diet (total score) [median (IQR)]	8.00 (7.00, 9.00)	8.00 (7.00, 9.00)	8.00 (7.00, 10.00)	0.003
Biochemical parameters				
Troponin I (ng/mL) [median (IQR)]	4.84 (3.00, 7.91)	3.98 (3.00, 6.07)	9.32 (6.28, 13.66)	<0.001
NT-proBNP (pg/mL) [median (IQR)]	50.58 (26.33, 95.48)	42.08 (21.78, 75.01)	115.50 (56.52, 221.02)	<0.001
hs-CRP (mg/dL) [median (IQR)]	0.12 (0.06, 0.27)	0.10 (0.06, 0.24)	0.16 (0.10, 0.35)	<0.001
Creatinine (mg/dL) [median (IQR)]	0.82 (0.71, 0.94)	0.81 (0.71, 0.93)	0.87 (0.75, 1.05)	<0.001
Ion chloride (mmol/L) [median (IQR)]	103.00 (101.00, 105.00)	103.00 (101.00, 105.00)	102.00 (100.00, 104.00)	0.001
Ion sodium (mmol/L) [median (IQR)]	141.00 (140.00, 143.00)	141.00 (140.00, 143.00)	141.00 (139.00, 143.00)	0.615
AST (U/L) [median (IQR)]	18.00 (16.00, 22.00)	18.00 (15.00, 21.00)	19.00 (16.00, 24.00)	0.001
ALT (U/L) [median (IQR)]	16.00 (11.00, 22.00)	16.00 (11.00, 22.00)	16.00 (12.00, 21.00)	0.540
Iron (µg/dL) [median (IQR)]	88.00 (70.00, 111.00)	88.00 (70.00, 111.00)	86.00 (68.00, 111.00)	0.177
Ferritin (ng/mL) [median (IQR)]	132.00 (59.00, 240.00)	129.00 (57.00, 240.00)	144.00 (66.50, 240.50)	0.350
Transferrin (mg/mL) [median (IQR)]	246.00 (225.00, 270.00)	246.00 (226.00, 268.00)	245.50 (224.00, 279.50)	0.647
Glycosylated hemoglobin (mmol/L) [median (IQR)]	5.40 (5.20, 5.60)	5.30 (5.10, 5.50)	5.60 (5.40, 5.90)	<0.001

ALT: alanine aminotransferase; AST: aspartate transaminase; BMI: body mass index; bpm: beats per minute; COPD: chronic obstructive pulmonary disease; hs-CRP: high-sensitivity C-reactive protein; DBP: diastolic blood pressure; HDL: high-density lipoprotein; IQR: interquartile range; LDL: low-density lipoprotein; NT-proBNP: N-terminal pro-brain natriuretic peptide; SBP: systolic blood pressure; TIA: transient ischemic attack; U: units. Values are presented as median [IQR] or n (%). Between-group comparisons were performed using Mann–Whitney U tests, or chi-square tests, as appropriate.

**Table 2:** Multivariate analysis of the association between plasma GDF15 group (≤1081 pg/mL vs. >1081 pg/mL) and heart diseases; the low category (≤1081) was used as reference (OR = 1.00).

Plasma GDF15 (pg/mL)									
	All Individuals			Women			Men		
	≤1081	>1081		≤1081	>1081		≤1081	>1081	
Model	Reference	OR (95% CI)	HL Test (χ^2^, df, *p*-Value)	Reference	OR (95% CI)	HL Test (χ^2^, df, *p*-Value)	Reference	OR (95% CI)	HL Test (χ^2^, df, *p*-Value)
(A) Heart failure									
Unadjusted	1.00	2.86 (1.7–4.82) ***	-	1.00	3.74 (1.42–9.85) *	-	1.00	2.29 (1.23–4.27) **	-
Model 1	1.00	1.97 (1.03–3.78) *	2.54, 8, 0.96	1.00	1.51 (0.48–4.8)	3.12, 8, 0.927	1.00	2.27 (1.03–4.98) *	17.39, 8, 0.026
Model 2	1.00	2 (0.85–4.71)	7.47, 8, 0.487	1.00	3.22 (0.65–15.91)	8.57, 8, 0.38	1.00	1.57 (0.55–4.45)	4.7, 8, 0.789
(B) Ischemic cardiomyopathy									
Unadjusted	1.00	5.85 (3.25–10.53) ***	-	1.00	2.78 (0.98–7.93).	-	1.00	7.75 (3.63–16.57) ***	-
Model 1	1.00	2.43 (1.2–4.91) *	10.68, 8, 0.221	1.00	1.04 (0.31–3.55)	11.15, 8, 0.193	1.00	3.61 (1.47–8.88) **	5.65, 8, 0.686
Model 2	1.00	3.34 (1.38–8.11) **	10.19, 8, 0.252	1.00	2.11 (0.47–9.55)	6.47, 8, 0.595	1.00	4.26 (1.4–12.96) *	12.27, 8, 0.139
(C) Arrhythmias									
Unadjusted	1.00	4.66 (3.11–6.98) ***	-	1.00	4 (2.1–7.63) ***	-	1.00	4.79 (2.83–8.12) ***	-
Model 1	1.00	1.62 (0.99–2.67)	10.7, 8, 0.219	1.00	1.74 (0.79–3.82)	14.3, 8, 0.074	1.00	1.53 (0.8–2.94)	3.41, 8, 0.906
Model 2	1.00	1.52 (0.81–2.87)	6.34, 8, 0.609	1.00	1.36 (0.47–3.94)	4.84, 8, 0.775	1.00	1.67 (0.74–3.76)	6.4, 8, 0.603
(D) Valvulopathy									
Unadjusted	1.00	2.25 (1.4–3.61) ***	-	1.00	1.88 (0.64–5.47)	-	1.00	4.45 (1.56–12.68) **	-
Model 1	1.00	1.87 (0.76–4.63)	9.91, 8, 0.271	1.00	1.23 (0.34–4.4)	9.11, 8, 0.333	1.00	3.02 (0.76–11.96)	7.76, 8, 0.457
Model 2	1.00	1.48 (0.46–4.69)	5.28, 8, 0.727	1.00	0.62 (0.11–3.67)	8.82, 8, 0.358	1.00	3.5 (0.57–21.32)	6.13, 8, 0.632

Unadjusted model: included GDF15 as the sole independent variable. Model 1: age, sex, current smoking, daily alcohol consumption and BMI. Model 2 (fully adjusted): Model 1 plus NT-proBNP. OR: odds ratio. HL: Hosmer–Lemeshow. df: degrees of freedom. *** *p* < 0.001 vs. reference; ** *p* < 0.01 vs. reference; * *p* < 0.05 vs. reference.

**Table 3:** Cause of death during follow-up according to GDF15 group and sex.

Cause of Death	GDF15 (pg/mL)				
		≤1081	>1081	≤1081	>1081
	Overall	Women n (%)		Men n (%)	
Neoplasms	17 (33.3)	4 (23.5)	2 (11.7)	3 (8.8)	8 (23.5)
Diseases of the circulatory system	13 (25.5)	-	5 (29.4)	3 (8.8)	5 (14.7)
Diseases of the respiratory system	5 (9.8)	1 (5.8)	-	1 (2.9)	3 (8.8)
COVID-19	3 (5.9)	-	-	-	3 (8.8)
Diseases of the genitourinary system	2 (3.9)	-	1 (5.8)	-	1 (2.9)
Vehicle occupant injured in transport accident	2 (3.9)	1 (5.8)	-	-	1 (2.9)
Slips, trips, and falls	2 (3.9)	-	-	1 (2.9)	1 (2.9)
Mental, behavioral and neurodevelopmental disorders	2 (3.9)	-	1 (5.8)	-	1 (2.9)
Inhalation/ingestion of foreign object causing airway obstruction	1 (2.0)	-	-	-	1 (2.9)
Diseases of the skin and subcutaneous tissue	1 (2.0)	-	1 (5.8)	-	-
Diseases of the digestive system	1 (2.0)	-	1 (5.8)	-	-
Diseases of the nervous system	1 (2.0)	-	-	-	1 (2.9)
Endocrine, nutritional, and metabolic diseases	1 (2.0)	-	-	-	1 (2.9)
Total	51 (100)	6 (35.3)	11 (64,7)	8 (23.5)	26 (76.5)

**Table 4:** Discriminative performance of Cox regression models for all-cause mortality.

Model	Variables Included	n; Number of Deaths	C-Index	ΔC-Index	LRT χ^2^	*p*-Value
Base model	age, sex, current smoking, daily alcohol consumption, BMI, heart disease	1522; 50	0.839	—	—	—
Base model + GDF15	age, sex, current smoking, daily alcohol consumption, BMI, heart disease, GDF15	1522; 50	0.849	0.01	6.38	0.011
Base model	age, sex, current smoking, daily alcohol consumption, BMI, heart disease	1020; 34	0.879	—	—	—
Base model + NT-proBNP	age, sex, current smoking, daily alcohol consumption, BMI, heart disease, NT-proBNP	1020; 34	0. 0.88	0.001	3.7635	0.052

Base model: age, sex, smoking, alcohol, BMI, and prevalent heart disease. BMI: body mass index. Concordance index (C-index) and Likelihood Ratio Test (LRT) results are shown.

## Data Availability

The data presented in this study are available on request from the corresponding authors.

## Supplementary Materials

The following supporting information can be downloaded at: https://www.mdpi.com/article/10.3390/ijms27073078/s1.

## Abbreviations

The following abbreviations are used in this manuscript:

ALT	Alanine aminotransferase
AST	Aspartate transaminase
BMI	Body mass index
COPD	Chronic obstructive pulmonary disease
GDNF	Glial-derived neurotrophic factor
GFRAL	Glial-derived neurotrophic factor receptor alpha-like
HbA1c	Glycated Haemoglobin
hs-CRP	High-sensitivity C-reactive protein
hs-TnT	High-sensitivity Troponin T
GDF15	Growth differentiation factor 15
LDL	Low density lipoprotein
MIC-1	Macrophage inhibitory cytokine-1
NT-proBNP	N-terminal pro-brain natriuretic peptide
TGF-β	Transforming growth factor-β
TIA	Transient ischemic attack

## References

[1] Timmis A., Vardas P., Townsend N., Torbica A., Katus H., De Smedt D., Gale C.P., Maggioni A.P., Petersen S.E., Huculeci R. (2022). European Society of Cardiology: Cardiovascular disease statistics 2021. Eur. Heart J..

[2] Vaduganathan M., Mensah G.A., Turco J.V., Fuster V., Roth G.A. (2022). The Global Burden of Cardiovascular Diseases and Risk: A Compass for Future Health. J. Am. Coll. Cardiol..

[3] Bootcov M.R., Bauskin A.R., Valenzuela S.M., Moore A.G., Bansal M., He X.Y., Zhang H.P., Donnellan M., Mahler S., Pryor K. (1997). MIC-1, a novel macrophage inhibitory cytokine, is a divergent member of the TGF-beta superfamily. Proc. Natl. Acad. Sci. USA.

[4] Li J., Hu X., Xie Z., Li J., Huang C., Huang Y. (2024). Overview of growth differentiation factor 15 (GDF15) in metabolic diseases. Biomed. Pharmacother..

[5] Wischhusen J., Melero I., Fridman W.H. (2020). Growth/Differentiation Factor-15 (GDF-15): From Biomarker to Novel Targetable Immune Checkpoint. Front. Immunol..

[6] Meijers W.C., Bayes-Genis A., Mebazaa A., Bauersachs J., Cleland J.G.F., Coats A.J.S., Januzzi J.L., Maisel A.S., McDonald K., Mueller T. (2021). Circulating heart failure biomarkers beyond natriuretic peptides: Review from the Biomarker Study Group of the Heart Failure Association (HFA), European Society of Cardiology (ESC). Eur. J. Heart Fail..

[7] Silva-Bermudez L.S., Klüter H., Kzhyshkowska J.G. (2024). Macrophages as a Source and Target of GDF-15. Int. J. Mol. Sci..

[8] Packer M. (2025). The Adipokine Hypothesis of Heart Failure with a Preserved Ejection Fraction: A Novel Framework to Explain Pathogenesis and Guide Treatment. J. Am. Coll. Cardiol..

[9] Wang T., Liu J., McDonald C., Lupino K., Zhai X., Wilkins B.J., Hakonarson H., Pei L. (2017). GDF15 is a heart-derived hormone that regulates body growth. EMBO Mol. Med..

[10] Conte M., Giuliani C., Chiariello A., Iannuzzi V., Franceschi C., Salvioli S. (2022). GDF15, an emerging key player in human aging. Ageing Res. Rev..

[11] Zhou Z., Liu H., Ju H., Chen H., Jin H., Sun M. (2023). Circulating GDF-15 in relation to the progression and prognosis of chronic kidney disease: A systematic review and dose-response meta-analysis. Eur. J. Intern. Med..

[12] Tian T., Liu M., Little P.J., Strijdom H., Weng J., Xu S. (2025). Emerging Roles of GDF15 in Metabolic and Cardiovascular Diseases. Research.

[13] Salminen A. (2024). GDF15/MIC-1: A stress-induced immunosuppressive factor which promotes the aging process. Biogerontology.

[14] Joo M., Kim D., Lee M.W., Lee H.J., Kim J.M. (2023). GDF15 Promotes Cell Growth, Migration, and Invasion in Gastric Cancer by Inducing STAT3 Activation. Int. J. Mol. Sci..

[15] Wada H., Suzuki M., Matsuda M., Ajiro Y., Shinozaki T., Sakagami S., Yonezawa K., Shimizu M., Funada J., Takenaka T. (2020). Impact of Smoking Status on Growth Differentiation Factor 15 and Mortality in Patients with Suspected or Known Coronary Artery Disease: The ANOX Study. J. Am. Heart Assoc..

[16] Mullican S.E., Lin-Schmidt X., Chin C.N., Chavez J.A., Furman J.L., Armstrong A.A., Beck S.C., South V.J., Dinh T.Q., Cash-Mason T.D. (2017). GFRAL is the receptor for GDF15 and the ligand promotes weight loss in mice and nonhuman primates. Nat. Med..

[17] Lasaad S., Crambert G. (2024). GDF15, an Emerging Player in Renal Physiology and Pathophysiology. Int. J. Mol. Sci..

[18] Rochette L., Zeller M., Cottin Y., Vergely C. (2020). Insights Into Mechanisms of GDF15 and Receptor GFRAL: Therapeutic Targets. Trends Endocrinol. Metab..

[19] Wollert K.C., Kempf T., Wallentin L. (2017). Growth Differentiation Factor 15 as a Biomarker in Cardiovascular Disease. Clin. Chem..

[20] Wang Z., Yang F., Ma M., Bao Q., Shen J., Ye F., Xie X. (2020). The impact of growth differentiation factor 15 on the risk of cardiovascular diseases: Two-sample Mendelian randomization study. BMC Cardiovasc. Disord..

[21] Wesseling M., de Poel J.H.C., de Jager S.C.A. (2020). Growth differentiation factor 15 in adverse cardiac remodelling: From biomarker to causal player. ESC Heart Fail..

[22] Wang J., Wei L., Yang X., Zhong J. (2019). Roles of Growth Differentiation Factor 15 in Atherosclerosis and Coronary Artery Disease. J. Am. Heart Assoc..

[23] Nopp S., Konigsbrugge O., Kraemmer D., Pabinger I., Ay C. (2021). Growth differentiation factor-15 predicts major adverse cardiac events and all-cause mortality in patients with atrial fibrillation. Eur. J. Intern. Med..

[24] Takaoka M., Tadross J.A., Al-Hadithi A., Zhao X., Villena-Gutierrez R., Tromp J., Absar S., Au M., Harrison J., Coll A.P. (2024). GDF15 antagonism limits severe heart failure and prevents cardiac cachexia. Cardiovasc. Res..

[25] Kempf T., Sinning J.M., Quint A., Bickel C., Sinning C., Wild P.S., Schnabel R., Lubos E., Rupprecht H.J., Munzel T. (2009). Growth-differentiation factor-15 for risk stratification in patients with stable and unstable coronary heart disease: Results from the AtheroGene study. Circ. Cardiovasc. Genet..

[26] Sakamoto D., Matsuoka Y., Nakatani D., Okada K., Sunaga A., Kida H., Sato T., Kitamura T., Tamaki S., Seo M. (2025). Role and prognostic value of growth differentiation factor 15 in patient of heart failure with preserved ejection fraction: Insights from the PURSUIT-HFpEF registry. Open Heart.

[27] Wang J., Han L.N., Ai D.S., Wang X.Y., Zhang W.J., Xu X.R., Liu H.B., Zhang J., Wang P., Li X. (2023). Growth differentiation factor 15 predicts cardiovascular events in stable coronary artery disease. J. Geriatr. Cardiol..

[28] Fabiani I., Santoni T., Angelillis M., Petricciuolo S., Colli A., Pellegrini G., Mazzei D., Pugliese N.R., Petronio A.S., De Caterina R. (2020). Growth Differentiation Factor 15 in Severe Aortic Valve Stenosis: Relationship with Left Ventricular Remodeling and Frailty. J. Clin. Med..

[29] Li B., Shaikh F., Younes H., Abuhalimeh B., Zamzam A., Abdin R., Qadura M. (2025). Growth Differentiation Factor 15 Predicts Cardiovascular Events in Peripheral Artery Disease. Biomolecules.

[30] Melero-Alegria J.I., Cascon M., Romero A., Vara P.P., Barreiro-Perez M., Vicente-Palacios V., Perez-Escanilla F., Hernandez-Hernandez J., Garde B., Cascon S. (2019). SALMANTICOR study. Rationale and design of a population-based study to identify structural heart disease abnormalities: A spatial and machine learning analysis. BMJ Open.

[31] di Candia A.M., de Avila D.X., Moreira G.R., Villacorta H., Maisel A.S. (2021). Growth differentiation factor-15, a novel systemic biomarker of oxidative stress, inflammation, and cellular aging: Potential role in cardiovascular diseases. Am. Heart J. Plus.

[32] Chuang W.C., Chu C.H., Yao C.S., Wei M.C., Hsieh I.L., Liao C.M. (2025). The value of growth differentiation factor 15 as a biomarker for peripheral artery disease in diabetes patients. Diabetol. Metab. Syndr..

[33] Aguilar-Recarte D., Barroso E., Palomer X., Wahli W., Vazquez-Carrera M. (2022). Knocking on GDF15’s door for the treatment of type 2 diabetes mellitus. Trends Endocrinol. Metab..

[34] Wan Y., Fu J. (2024). GDF15 as a key disease target and biomarker: Linking chronic lung diseases and ageing. Mol. Cell. Biochem..

[35] Ozdemir E., Ziyrek M. (2025). Effect of growth differentiation factor-15 (GDF-15) level on extent and severity of atherosclerosis in peripheral arterial disease patients free of obstructive coronary artery disease: A cross-sectional observational study. Medicine.

[36] Liu H., Huang Y., Lyu Y., Dai W., Tong Y., Li Y. (2021). GDF15 as a biomarker of ageing. Exp. Gerontol..

[37] Oba K., Ishikawa J., Tamura Y., Fujita Y., Ito M., Iizuka A., Fujiwara Y., Kodera R., Toyoshima K., Chiba Y. (2024). Serum Growth Differentiation Factor 15 Levels Predict the Incidence of Frailty among Patients with Cardiometabolic Diseases. Gerontology.

[38] Binder M.S., Yanek L.R., Yang W., Butcher B., Norgard S., Marine J.E., Kolandaivelu A., Chrispin J., Fedarko N.S., Calkins H. (2023). Growth Differentiation Factor-15 Predicts Mortality and Heart Failure Exacerbation But Not Ventricular Arrhythmias in Patients with Cardiomyopathy. J. Am. Heart Assoc..

[39] Hullwegen M., Kleinert M., von Haehling S., Fischer A. (2025). GDF15: From biomarker to target in cancer cachexia. Trends Cancer.

[40] Javaheri A., Ozcan M., Moubarak L., Smoyer K.E., Rossulek M.I., Revkin J.H., Groarke J.D., Tarasenko L.C., Kosiborod M.N. (2024). Association between growth differentiation factor-15 and adverse outcomes among patients with heart failure: A systematic literature review. Heliyon.

[41] Sharma A., Stevens S.R., Lucas J., Fiuzat M., Adams K.F., Whellan D.J., Donahue M.P., Kitzman D.W., Pina I.L., Zannad F. (2017). Utility of Growth Differentiation Factor-15, A Marker of Oxidative Stress and Inflammation, in Chronic Heart Failure: Insights from the HF-ACTION Study. JACC Heart Fail..

[42] Berezin A.E. (2016). Diabetes mellitus related biomarker: The predictive role of growth-differentiation factor-15. Diabetes Metab. Syndr..

[43] Huang X., Pan C.H., Yin F., Peng J., Yang L. (2025). The Role of Estrogen in Mitochondrial Disease. Cell. Mol. Neurobiol..

[44] Gayet-Ageron A., Rudaz S., Perneger T. (2016). Biobank attributes associated with higher patient participation: A randomized study. Eur. J. Hum. Genet..

[45] Wong M.L., Chia K.S., Yam W.M., Teodoro G.R., Lau K.W. (2004). Willingness to donate blood samples for genetic research: A survey from a community in Singapore. Clin. Genet..

[46] Martinez-Camblor P., Pardo-Fernandez J.C. (2019). The Youden Index in the Generalized Receiver Operating Characteristic Curve Context. Int. J. Biostat..

