# Supplementary material for: Growth Differentiation Factor 15 as a Biomarker of Cardiovascular Burden and Mortality in a Population-Based Cohort
Source: Int J Mol Sci. 2026 Mar 27;27(7):3078. doi: 10.3390/ijms27073078 (PMC13073294; doi:10.3390/ijms27073078)
Supplement: Supplementary file 1 [file ijms-27-03078-s001.zip › Table S2.pdf]

Table S2. Multivariate analysis of the association between demographic, lifestyle, clinical, and biomarker variables and mortality; the GDF15 ≤1081 pg/mL category was used as the reference (HR = 1.00).

	**Base model**	**Model A**	**Model B**	**Model C**
**Age**	4.17 [2.7–6.43] ***	3.04 [1.86–4.96] ***	4.59 [2.32–9.08] ***	6.3 [3.42–11.62] ***
**Sex: men**	2.94 [1.59–5.43] ***	2.69 [1.45–4.98] **	2.79 [1.29–6.02] **	3.07 [1.43–6.61] **
**Current smoking**	2.4 [0.97–5.9]	2.09 [0.84–5.19]	4.51 [1.57–12.93] **	4.91 [1.74–13.89] **
**Daily alcohol consumption**	0.46 [0.24–0.89] *	0.5 [0.25–0.97] *	0.53 [0.23–1.23]	0.49 [0.21–1.12]
**BMI**	0.9 [0.65–1.26]	0.88 [0.64–1.22]	1.22 [0.84–1.77]	1.24 [0.86–1.79]
**Heart disease**	2.72 [1.49–4.96] ***	2.64 [1.45–4.79] **	1.98 [0.92–4.28]	2.1 [0.97–4.56]
**GDF15 > 1081 pg/mL**	-	2.47 [1.19–5.13] *	2.61 [0.99–6.87] .	-
**NT-proBNP**	-	-	1.11 [1–1.22] *	1.12 [1.02–1.23] *

Hazard ratios (HR) with 95% confidence intervals (CI) are shown for demographic, lifestyle, clinical, and biomarker variables. Multivariate adjustments; Base model: age, sex, current smoking, daily alcohol consumption, BMI, and prevalent heart disease; Model A: Base model + GDF15; Model B: Model A + NT-proBNP; Model C: Base model + NT-proBNP. ***p < 0.001; **p < 0.01; *p < 0.05; p < 0.1.
