# Supplementary material for: Growth Differentiation Factor 15 as a Biomarker of Cardiovascular Burden and Mortality in a Population-Based Cohort
Source: Int J Mol Sci. 2026 Mar 27;27(7):3078. doi: 10.3390/ijms27073078 (PMC13073294; doi:10.3390/ijms27073078)
Supplement: Supplementary file 1 [file ijms-27-03078-s001.zip › Table S1.pdf]

Table S1. Association between elevated GDF15 and prevalent heart disease: logistic regression analyses overall and by sex.

A) All individuals

Base model

	Heart failure	Ischemic cardiomyopathy	Arrhythmias	Valvulopathy
**Age**	1.55[1.16-2.06] **	2.95[2-4.33] ***	2.98[2.29-3.88] ***	1.9[1.28-2.82] ***
**Sex: Men**	3.61[1.98-6.59] ***	2.43[1.24-4.78] **	2.33[1.47-3.68] ***	1.55[0.72-3.32]
**Current smoking**	0.97[0.47-2]	2.04[0.93-4.45]	0.69[0.33-1.43]	0.59[0.17-2.04]
**Daily alcohol consumption**	0.59[0.32-1.08]	1.23[0.65-2.33]	0.76[0.47-1.23]	0.47[0.18-1.23]
**BMI**	1.21[0.95-1.54]	1.34[1.01-1.76] *	1.15[0.92-1.43]	0.69[0.44-1.07]

Model 1

	Heart failure	Ischemic cardiomyopathy	Arrhythmias	Valvulopathy
**Age**	1.27[0.9-1.78]	2.19[1.4-3.42] ***	2.53[1.85-3.45] ***	1.58[0.99-2.53]
**Sex: Men**	3.44[1.88-6.28] ***	2.22[1.13-4.39] *	2.21[1.39-3.5] ***	1.45[0.67-3.13]
**Current smoking**	0.93[0.45-1.91]	1.91[0.87-4.21]	0.66[0.32-1.37]	0.56[0.16-1.93]
**Daily alcohol consumption**	0.61[0.33-1.14]	1.33[0.7-2.53]	0.8[0.5-1.3]	0.51[0.19-1.33]
**BMI**	1.21[0.95-1.54]	1.32[0.99-1.74]	1.13[0.91-1.41]	0.68[0.44-1.06]
**GDF15 > 1081**	1.97[1.03-3.78] *	2.43[1.2-4.91] *	1.62[0.99-2.67] .	1.87[0.76-4.63]

Model 2

	Heart failure	Ischemic cardiomyopathy	Arrhythmias	Valvulopathy
**Age**	1.35[0.82-2.21]	1.63[0.95-2.79]	1.94[1.31-2.86] ***	1.88[0.99-3.58]
**Sex: Men**	3.16[1.43-7] **	1.82[0.8-4.17]	1.83[1.02-3.29] *	1.29[0.49-3.41]
**Current smoking**	1.67[0.69-4.02]	2.26[0.91-5.66]	0.64[0.26-1.58]	0.36[0.05-2.81]
**Daily alcohol consumption**	0.58[0.25-1.33]	1.49[0.67-3.33]	1.21[0.67-2.19]	0.8[0.26-2.45]
**BMI**	1.41[1.09-1.83] **	1.28[0.93-1.77]	1.2[0.93-1.55]	0.73[0.42-1.25]
**GDF15 > 1081**	2[0.85-4.71]	3.34[1.38-8.11] **	1.52[0.81-2.87]	1.48[0.46-4.69]
**NT-proBNP**	1.33[1.08-1.63] **	1.14[0.96-1.35]	1.54[1.25-1.9] ***	1.23[1.02-1.48] *

B) Women

Base model

	Heart failure	Ischemic cardiomyopathy	Arrhythmias	Valvulopathy
**Age**	2.59[1.4-4.79] **	2.56[1.32-4.97] **	2.41[1.62-3.58] ***	1.84[1.04-3.24] *
**Current smoking**	2.3[0.57-9.24]	4.08[1.08-15.47] *	0.75[0.22-2.6]	0.88[0.19-4.18]
**Daily alcohol consumption**	0.71[0.16-3.21]	0.83[0.18-3.81]	0.99[0.4-2.48]	0.8[0.17-3.62]
**BMI**	1.21[0.74-1.97]	1.4[0.86-2.27]	1.14[0.82-1.6]	0.59[0.32-1.11]

Model 1

	Heart failure	Ischemic cardiomyopathy	Arrhythmias	Valvulopathy
**Age**	2.3[1.15-4.61] *	2.53[1.21-5.31] *	2.05[1.3-3.23] **	1.75[0.91-3.33]
**Current smoking**	2.23[0.55-9.05]	4.07[1.07-15.48] *	0.7[0.2-2.45]	0.86[0.18-4.12]
**Daily alcohol consumption**	0.75[0.16-3.42]	0.83[0.18-3.84]	1.1[0.43-2.79]	0.82[0.18-3.77]
**BMI**	1.19[0.73-1.92]	1.39[0.85-2.27]	1.12[0.8-1.56]	0.59[0.32-1.11]
**GDF15 > 1081**	1.51[0.48-4.8]	1.04[0.31-3.55]	1.74[0.79-3.82]	1.23[0.34-4.4]

Model 2

	Heart failure	Ischemic cardiomyopathy	Arrhythmias	Valvulopathy
**Age**	1.61[0.63-4.12]	2.29[0.9-5.85]	1.45[0.83-2.52]	1.87[0.77-4.53]
**Current smoking**	3.15[0.51-19.34]	5.62[1.12-28.29]	0.33[0.04-2.6]	0.95[0.11-8.47]
**Daily alcohol consumption**	0.83[0.1-7.11]	0.58[0.07-4.81]	2.15[0.75-6.21]	1.2[0.21-6.93]
**BMI**	1.62[0.96-2.75]	1.07[0.58-1.97]	1.26[0.85-1.89]	0.55[0.25-1.21]
**GDF15 > 1081**	1.18[0.87-1.61]	2.11[0.47-9.55]	1.36[0.47-3.94]	0.62[0.11-3.67]
**NT-proBNP**	3.22[0.65-15.91]	1.11[0.79-1.56]	1.63[1.25-2.13] ***	1.54[1.16-2.06] **

C) Men

Base model

	Heart failure	Ischemic cardiomyopathy	Arrhythmias	Valvulopathy
**Age**	1.3[0.93-1.81]	3.28[2.01-5.36] ***	3.58[2.47-5.17] ***	2.08[1.17-3.68] *
**Current smoking**	0.76[0.33-1.78]	1.5[0.57-3.97]	0.66[0.27-1.65]	0.36[0.05-2.89]
**Daily alcohol consumption**	0.6[0.31-1.17]	1.34[0.65-2.76]	0.68[0.39-1.18]	0.35[0.11-1.14]
**BMI**	1.19[0.89-1.58]	1.35[0.96-1.91]	1.21[0.9-1.62]	0.86[0.45-1.66]

OR[CI 95%]. *** p < 0.001; ** p < 0.01; * p < 0.05.

Model 1

	Heart failure	Ischemic cardiomyopathy	Arrhythmias	Valvulopathy
**Age**	1.02[0.68-1.52]	2.1[1.19-3.68] **	3.06[1.98-4.72] ***	1.44[0.71-2.93]
**Current smoking**	0.72[0.31-1.68]	1.39[0.52-3.74]	0.64[0.26-1.6]	0.32[0.04-2.6]
**Daily alcohol consumption**	0.63[0.32-1.23]	1.45[0.7-3.02]	0.7[0.4-1.22]	0.38[0.12-1.25]
**BMI**	1.19[0.89-1.59]	1.34[0.93-1.93]	1.2[0.89-1.61]	0.85[0.44-1.64]
**GDF15 > 1081**	2.27[1.03-4.98] *	3.61[1.47-8.88] **	1.53[0.8-2.94]	3.02[0.76-11.96]

Model 2

	Heart failure	Ischemic cardiomyopathy	Arrhythmias	Valvulopathy
**Age**	1.22[0.68-2.21]	1.46[0.73-2.9]	2.56[1.45-4.51] ***	1.69 [0.66-4.97
**Current smoking**	1.44[0.53-3.91]	1.6[0.52-4.93]	0.81[0.29-2.26]	0.26 [0-2.25]
**Daily alcohol consumption**	0.59[0.24-1.46]	1.8[0.71-4.57]	0.92[0.46-1.85]	0.47 [0.11-1.7]
**BMI**	1.34[0.99-1.82]	1.4[0.96-2.06]	1.26[0.9-1.76]	1.19 [0.44-1.97]
**GDF15 > 1081**	1.57[0.55-4.45]	4.26[1.4-12.96] *	1.67[0.74-3.76]	3.05 [0.6-19.28]
**NT-proBNP**	1.52[1.04-2.23] *	1.16[0.94-1.42]	1.46[0.99-2.15]	1.11 [0.89-1.34]

Logistic regression models examining the association between plasma GDF15 (≤1081 pg/mL vs. >1081 pg/mL) and prevalent heart failure, ischemic cardiomyopathy, arrhythmias, and valvulopathy. Analyses are shown for (A) all participants, (B) women, and (C) men. Base model: adjusted by age, sex, current smoking, daily alcohol consumption, and body mass index (BMI), but excluded GDF15 and NT-proBNP. Model 1: adjusted for age, sex, current smoking, daily alcohol consumption, BMI; Model 2-fully adjusted: model 1 plus NT-proBNP). Results are presented as odds ratios (OR) with 95% confidence intervals (CI). ***$p < 0.001$; **$p < 0.01$; *$p < 0.05$.
